# Association between hysterectomy, oophorectomy, and risk of breast cancer: a meta-analysis

**DOI:** 10.1007/s00404-025-08179-0

**Published:** 2025-11-29

**Authors:** Bing Wang, Meng Wang, Huili Xu, Yu Liu, Tengteng Kang, Yang Cao

**Affiliations:** 1 The First Hospital of Jilin University, Jilin, 130021 China; 2 Department of Nursing, Faculty of Medicine & Health Sciences, Universiti Putra Malaysia, 43400 UPM Serdang, Selangor Malaysia; 3 Xiangyang No.1 People’s Hospital, Hubei University of Medicine, Xiangyang, 441000 Hubei China; 4 Emergency Medicine Department of the Second Mobile Contingent Hospital of the Chinese People’s Armed Police Forces, Wuxi City, Jiangsu Province China; 5 Department of Nursing, Zhoukou Vocational and Technical College, Zhoukou, 466000 China; 6 Department of Nursing, Changchun University of Chinese Medicine, Changchun, 130117 Jilin China

**Keywords:** Hysterectomy, Oophorectomy, Breast cancer, Risk, Meta-analysis

## Abstract

**Objectives:**

This meta-analysis seeks to clarify the relationship between hysterectomy, oophorectomy, and the subsequent risk of developing breast cancer.

**Methods:**

A comprehensive literature search was conducted across PubMed, the Cochrane Library, and Embase to identify relevant studies. The quality of the included studies was assessed using the Newcastle–Ottawa Scale (NOS). Statistical analyses were performed using Stata software (version 14.0), with hazard ratios (HRs) and their corresponding 95% confidence intervals (CIs) calculated. Publication bias was assessed using funnel plots and Egger’s test.

**Results:**

A total of 12 studies were included, comprising 9 cohort studies and 3 case–control studies, with publication years ranging from 1988 to 2023, involving 5,868,660 participants, predominantly from the United States. The analysis revealed that both hysterectomy and oophorectomy are associated with a reduced risk of breast cancer, lowering the risk by 16% (HR 0.84; 95% CI: 0.76–0.92; *I*^*2*^ = 76.5%; *P* < 0.001). Standalone hysterectomy was associated with a 13% reduction in breast cancer risk (HR 0.87; 95% CI: 0.77–0.99; *I*^*2*^ = 82.3%; *P* = 0.033), while bilateral oophorectomy reduced the risk by approximately 19% (HR 0.81; 95% CI: 0.68–0.96; *I*^*2*^ = 61.7%; *P* = 0.016). In contrast, unilateral oophorectomy did not significantly affect the risk of breast cancer (HR 0.89; 95% CI: 0.71–1.11; *I*^*2*^ = 45.5%; *P* = 0.288). Patients who underwent bilateral oophorectomy and received hormone therapy experienced a 20% reduction in breast cancer risk (HR 0.80; 95% CI: 0.68–0.93; *I*^*2*^ = 38.5%; *P* = 0.005), whereas those who did not receive hormone therapy showed no significant risk reduction (HR 0.87; 95% CI: 0.69–1.10; *I*^*2*^ = 48.5%; *P* = 0.254). Premenopausal bilateral oophorectomy was associated with a 13% decrease in breast cancer incidence risk (HR 0.87; 95% CI: 0.79–0.96; *I*^*2*^ = 0%; *P* = 0.004), while postmenopausal bilateral oophorectomy had no significant impact (HR 0.95; 95% CI: 0.88–1.03; *I*^*2*^ = 1.2%; *P* = 0.196).

**Conclusions:**

This meta-analysis suggests that both hysterectomy and oophorectomy are significantly associated with a reduction in breast cancer risk. The effectiveness of bilateral oophorectomy appears to be modulated by hormone therapy and menopausal status. Further research is needed to clarify these associations and to explore the underlying biological mechanisms.

**Supplementary Information:**

The online version contains supplementary material available at 10.1007/s00404-025-08179-0.

## What does this study add to the clinical work

**Table:** 

This study adds to clinical practice by providing evidence that both hysterectomy and oophorectomy, particularly bilateral oophorectomy, are associated with a reduced risk of breast cancer. It highlights the potential impact of hormone therapy and menopausal status on the effectiveness of these surgical interventions in lowering breast cancer risk.	

## Introduction

Breast cancer is the most common tumor among women, with an annual incidence rate increasing from 0.6 to 1.0% [1]. According to global cancer statistics, it has become the most diagnosed cancer and the second leading cause of cancer-related deaths among women [1–3]. Hysterectomy is the most common non-obstetric surgery performed in women [4, 5], used to treat conditions, such as uterine fibroids, endometriosis, and uterine cancer [6–8]. Oophorectomy, primarily performed to treat ovarian tumors and inflammation, can be classified into bilateral and unilateral oophorectomy [9, 10]. It is often conducted alongside hysterectomy, potentially reducing the risk of disease progression [11, 12]. Research indicates that women undergoing hysterectomy or oophorectomy may face an increased risk of osteoporosis and cardiovascular diseases [13–15]. There is also evidence linking these procedures to a higher incidence of gynecological cancers [16, 17]. This association may result from decreased estrogen levels following hysterectomy or oophorectomy, affecting the reproductive system and bone density. Some studies suggest that women who undergo hysterectomy may be more susceptible to breast cancer [18], while others indicate that hysterectomy could reduce breast cancer incidence [19]. Additionally, while some scholars, such as Lovett, propose that oophorectomy offers a protective effect, other studies identify it as a risk factor for breast cancer [20]. Currently, there is a lack of consensus and clarity from existing meta-analyses on these issues. Therefore, this study aims to comprehensively review the available population-based evidence to clarify the relationship between hysterectomy, oophorectomy, and breast cancer risk, providing a foundational reference for future research and clinical practice.

## Methods

This meta-analysis was conducted following the PRISMA (Preferred Reporting Items for Systematic Reviews and Meta-Analyses) statement guidelines [21]. The protocol is registered in the International Prospective Register of Systematic Reviews under CRD42024589162.

### Data sources and searches

To ensure a comprehensive review of relevant cohort studies, we searched three major databases: PubMed, Cochrane Library, and Embase. Our search covered the inception of these databases up to August 25, 2024, without language restrictions, promoting inclusivity. We utilized a combination of medical subject headings (MeSH) and keywords, including hysterectomy, hysterectomy*, ovariectomy, oophorectom*, breast neoplasms, breast tumor*, breast cancer*, breast carcinoma*, and risk. Detailed search strategies for each database are provided in Supplementary Table 1. Additionally, we manually reviewed reference lists and relevant articles of all eligible studies to enhance the comprehensiveness of our findings.

### Eligibility criteria

We applied specific eligibility criteria to select relevant studies, which included: (1) cohort or case–control study design; (2) participants aged 18 years or older; and (3) investigations into the association between hysterectomy, oophorectomy, and breast cancer risk. We excluded reports, reviews, conference abstracts, animal trials, and studies with duplicate results. Studies lacking essential estimates, such as odds ratios (OR), relative risks (RR), or hazard ratios (HR) with corresponding 95% confidence intervals (CI), were also excluded. These criteria ensured the inclusion of high-quality studies that accurately addressed the relationship between hysterectomy or oophorectomy and breast cancer risk.

### Study selection

Two researchers (MW and YC) independently reviewed the literature using predetermined inclusion and exclusion criteria. They first eliminated duplicate articles, and then conducted an initial screening based on titles and abstracts, followed by a thorough review of full texts of potentially eligible articles. Discrepancies were resolved through discussion with a third researcher (BW).

### Data extraction

Two researchers (MW and YC) adhered to established data extraction protocols using pre-designed tables. Extracted data included details, such as first author, publication year, country, study design, follow-up duration, sample size, age range, data source, surgical approach, assessment method, subgroup analyses, adjustment for confounding factors, and NOS scores. Any discrepancies were resolved by consulting a third researcher (YL).

### Risk-of-bias assessment

We assessed the quality of cohort studies using the Newcastle–Ottawa Scale (NOS) [22], evaluating participant selection, group comparability, and outcome assessment. NOS scores range from 0 to 9 points, with six studies classified as moderate quality and the remainder as high quality.

### Statistical analysis

A meta-analysis was performed using a random-effects model, specifically the DerSimonian–Laird method [23], to compare the overall hazard ratio (HR) of breast cancer between individuals who underwent oophorectomy and/or hysterectomy and those who did not. The reference category consisted of individuals who had not undergone either procedure. When multiple adjusted estimates were available, the most comprehensive estimate was selected. Subgroup analyses were conducted based on hormone use (pre- and post-menopause). Heterogeneity across the studies was assessed using the *I*^*2*^ statistic. If *I*^*2*^ ≤ 50% and *P* > 0.1, a fixed-effects model was employed, indicating minimal heterogeneity. In cases where *I*^*2*^ > 50%, reflecting substantial heterogeneity, a random-effects model was applied. Sensitivity analyses were performed to assess the robustness of the overall results. Publication bias was evaluated using a funnel plot and Egger’s test [24]. All statistical analyses were conducted using Stata software version 14.0.

## Results

### Literature search

**Fig. 1:**
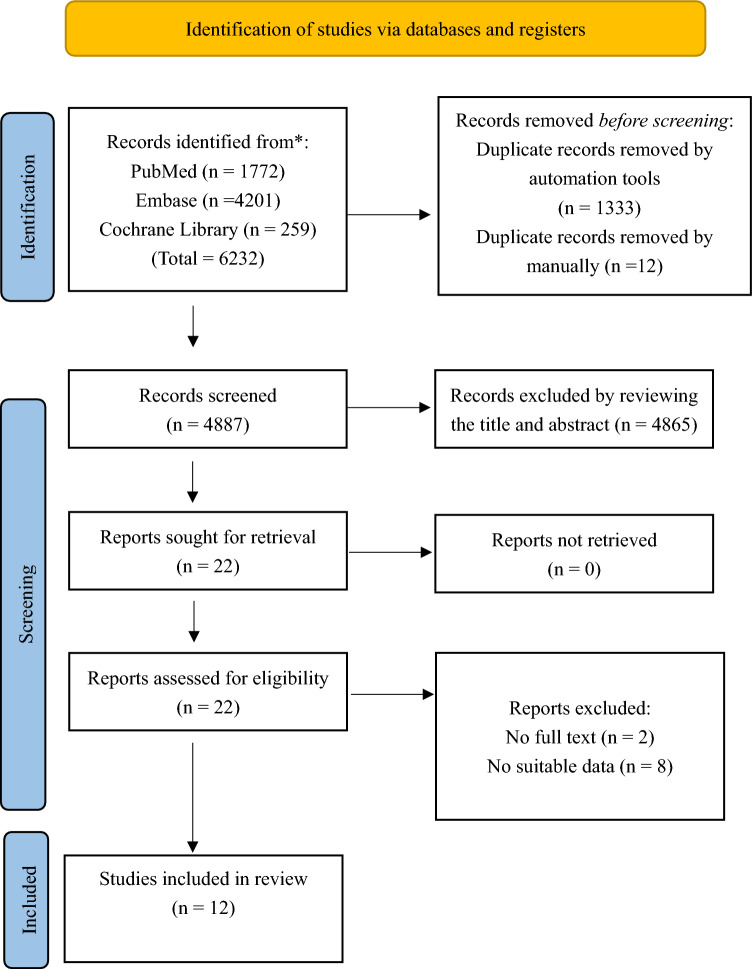
Flow diagram of study selection and exclusion

Figure 1 outlines the study selection process. From an initial pool of 6232 studies, 1333 duplicates were removed. After screening titles and abstracts, additional 4865 studies were excluded. Following a thorough full-text review, 10 more studies were eliminated, resulting in 12 studies that met the inclusion criteria for analysis.

### Characteristics of the eligible studies

This meta-analysis included 12 relevant studies—9 cohort studies and 3 case–control studies—published between 1988 and 2023, comprising a total of 5,868,660 participants. The studies were conducted across four countries, with the United States contributing the majority (8 studies) [18, 19, 25–30], followed by Denmark (2 studies) [31, 32], and one each from Italy and Sweden [20, 33]. All participants were aged 18 or older at the start of follow-up, which spanned from 3 to 36 years. The surgical procedures included oophorectomy, hysterectomy, or both, with disease assessments conducted via questionnaires, medical records, and interviews. Although methodologies for adjusting for confounding factors varied, nearly all studies reported adjusted estimates. Detailed characteristics of the included studies can be found in Table 1.

**Table 1:** Characteristics of the cohort and case-control studies included in the meta-analysis

Author years	Country	Design	Follow-up/data collect (years)	No. of participants	Age (years)	Data source	Type of surgery	Assessment tools	Subgroup analysis	NOS scores	Confounders adjusted
Irwin et al. [25]	American	Case–control study	3	Cases:4730 control:4688	20–54	Cancer and Steroid Hormone Study	Hysterectomy, Tubal sterilization	Interview	Age at surgery	7	Adjusted for age, data collection center, parity, family history of breast cancer, and history of benign breast disease
Parazzini et al. [20]	Italian	Case–control study	22	Cases:5984; control:5504	22–74;20–74	teaching and general hospitals	Hysterectomy, Oophorectomy	Medical record	Time since operation	8	Multivariate estimates, including terms for age, calendar year at interview, study, and center
Olson et al. [26]	American	Cohort study	24	680	< 60	Mayo Clinic Surgical Index	Bilateral oophorectomy	Telephone medical records	Age of surgery	7	/
Boggs et al. [27]	American	Cohort study	16	44,514	21–69	Black Women's Health Study	Hysterectomy; bilateral oophorectomy	Questionnaire	Age at surgery	7	Adjusted for age, BMI in 1995, menopausal hormone use, smoking status, educational attainment, geographic region, and family history of breast cancer
Gaudet et al. [28]	American	Cohort study	13.9	66,802	50–74	Cancer Prevention Study-II Nutrition Cohort	Hysterectomy, oophorectomy	Questionnaire	/	8	Multivariable-adjusted models controlled for attained age, race, education, alcohol consumption, smoking, parity, age at first birth, use of hormone replacement therapy, physical activity, age at menopause, and body mass index
Altman et al. [33]	Sweden	Cohort study	36	5,491,438	16–74	nationwide health-care registers	Bilateral salpingo-oophorectomy (BSO); hysterectomy	Medical record	Exposure period	8	/
Robinson et al. [19]	American	Case–control study	7	Cases:1664 control:1454	20–49	e Carolina Breast Cancer Study (CBCS)	Hysterectomy, Oophorectomy	Questionnaire; interviews	different races	7	Adjusted for age, squared age, race (when appropriate), family history of breast cancer, alcohol consumption, age at menarche, parity and age At first pregnancy composite, lactation history (ever vs. never breastfed), educational level, and smoking. Complete-case analysis was restricted to those with nonmissing values for these covariates
Terry et al. [29]	American	Cohort	10.7 years	17 917	18–79	Breast Cancer Family Registry (BCFR); the Kathleen Cunningham Foundation Consortium for Research into Familial Breast Cancer (kConFab)	Salpingo-oophorectomy	Medical record	/	8	/
Koch et al. [31]	Denmark	Cohort	25	24 409	≥ 45	Danish Nurse Organization	Oophorectomy; hysterectomy	Questionnaire	Age, BMI, HRT	8	Adjustment for age at menarche, oral contraceptives , parity, number of births, age at first birth, shift work, body mass index, smoking status, alcohol consumption, family history of breast/gynecological cancer, hormone replacement therapy, and hysterectomy
Huo et al. [30]	American	Cohort	18	1562	< 50	Rochester Epidemiology Project	Oophorectomy	Medical record	Age, ET, surgical indication	8	/
Gottschau et al. [32]	Denmark	Cohort	41	142 985	≥ 20	Danish Civil Registration System ; the Danish National Patient Register	Alpingo-oophorectomy; hysterectomy	Medical record	year	9	Adjusted for year of hysterectomy, age at hysterectomy, educational level, and comorbidity (diabetes, obesity, alcohol- and smoking related disease, hypertension and dyslipidemia);
Lovett et al. [18]	American	Cohort	11.4	50,701	35–74	Sister Study	Hysterectomy, oophorectomy	Telephone interview; questionnaires	Race and ethnicity; Family history of breast; ER status; BMI; Invasiveness	9	Adjusted for race and ethnicity, education , family history of breast cancer, alcohol consumption, smoking status, body mass index, hormonal birth control, age at menarche, parity, age at first pregnancy, breastfeeding , menopause status, and hormone therapy

### Quality assessment

All cohort studies were evaluated using the NOS scale, yielding an average score of 7.92 ± 0.67. Three studies received scores lower than 8 [31, 32], while the remaining studies scored 8 or higher, indicating a range from moderate to high methodological quality. The NOS assesses quality based on selection, comparability, and outcome assessment, with scores ranging from 0 to 9—higher scores signify greater research quality. Thus, the included studies exhibit robust methodological quality and reliable findings.

### Oophorectomy and/or hysterectomy and the risk of breast cancer

**Fig. 2:**
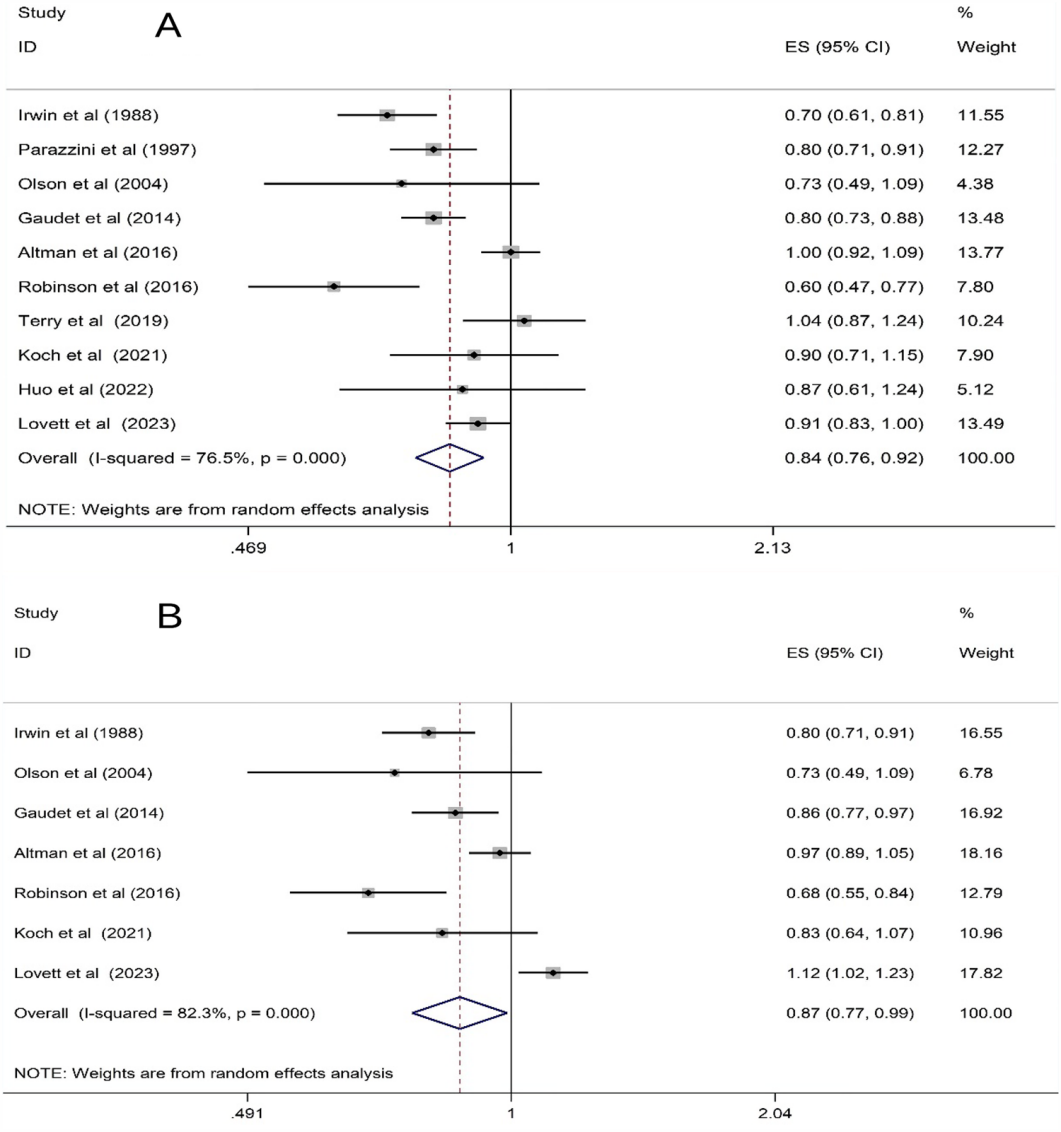
Forest plots for the association between hysterectomy, oophorectomy, and breast cancer risk

Ten studies were analyzed for the association between oophorectomy and/or hysterectomy and breast cancer risk [18–20, 25, 26, 28–31, 33]. Random-effects model analysis indicated that oophorectomy and hysterectomy were protective factors, reducing breast cancer risk by 16% compared to non-surgical patients (HR 0.84, 95% CI: 0.76–0.92; *I*^*2*^ = 76.5%; P < 0.001; Fig. 2A). Furthermore, isolated hysterectomy was associated with a 13% risk reduction (HR 0.87, 95% CI: 0.77–0.99; *I*^*2*^ = 82.3%; *P* = 0.033; Fig. 2B).

### Subgroup analysis

According to the subgroup analysis based on unilateral and bilateral oophorectomy, which included six studies [18–20, 27, 30, 31]. The results show that bilateral oophorectomy can effectively reduce the risk of breast cancer by approximately 19% (HR 0.81; 95% CI: 0.68–0.96; *I*^*2*^ = 61.7%; *P* = 0.016). Unilateral oophorectomy has no impact on the risk of breast cancer (HR, 0.89; 95% CI: 0.71–1.11; *I*^*2*^ = 45.5%; *P* = 0.288). Further research on patients who have undergone bilateral oophorectomy shows that postoperative hormone therapy can effectively reduce the risk of breast cancer by 20% (HR, 0.80; 95% CI: 0.68–0.93; *I*^*2*^ = 38.5%; *P* = 0.005), while not receiving hormone therapy has no observed impact on the risk of breast cancer (HR, 0.87; 95% CI: 0.69–1.10; *I*^*2*^ = 48.5%; *P* = 0.254). Additionally, we have also found that premenopausal bilateral oophorectomy reduces the risk of breast cancer by 13% (HR, 0.87; 95% CI: 0.79–0.96; *I*^*2*^ = 0%; *P* = 0.004), while postmenopausal bilateral oophorectomy has no effect on the risk of developing breast cancer (HR, 0.95; 95% CI: 0.88–1.03; *I*^*2*^ = 1.2%; *P* = 0.196), (Table 2).

**Table 2:** Subgroup analyses of oophorectomy and breast cancer risk

Subgroups	Included studies	OR (95%CI)	Heterogeneity	
			*I*^*2*^ (%)	*P* values
Oophorectomy				
Bilateral	5	0.81(0.68–0.96)	61.7	0.033
Unilateral	3	0.89(0.71–1.11)	45.5	0.160
Bilateral oophorectomy				
Use of hormones	3	0.80(0.68–0.93)	38.5	0.197
No use of hormones	3	0.87(0.69–1.10)	48.5	0.143
Premenopausal	4	0.87(0.79–0.96)	0	0.412
Postmenopausal	3	0.95(0.88–1.03)	1.2	0.363

### Publication bias

**Fig. 3:**
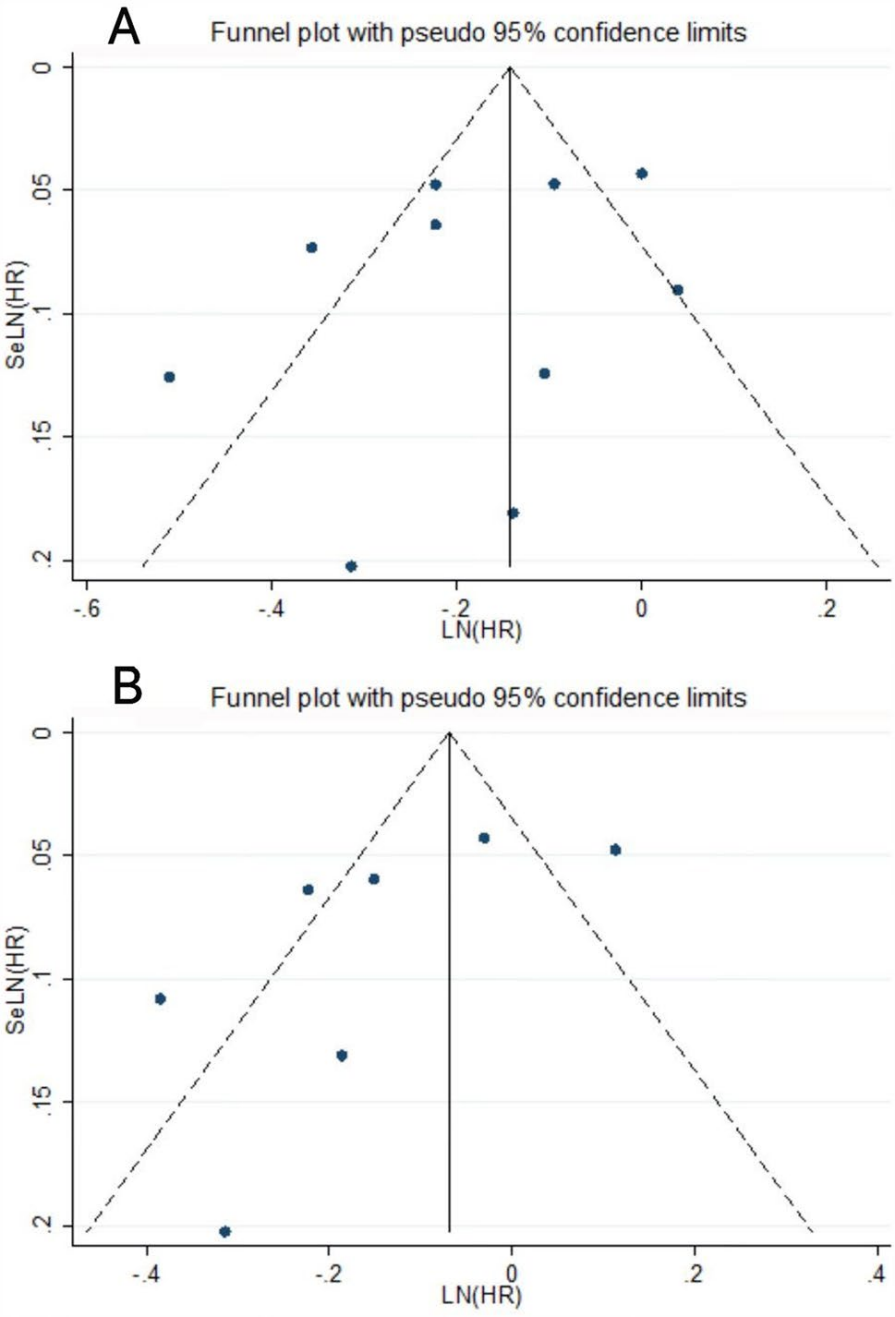
Funnel plot showing the effect of hysterectomy, oophorectomy, and breast cancer risk. **A** Hysterectomy and/or oophorectomy and risk of breast cancer. **B** Oophorectomy and breast cancer risk

Visual inspection of the funnel plot revealed no significant publication bias regarding the outcomes of oophorectomy and hysterectomy versus breast cancer risk. Egger’s regression test (*P* > 0.05) further supported the absence of publication bias in this meta-analysis (Fig. 3).

## Discussion

### Main findings

This meta-analysis consisted of three case–control studies and nine cohort studies, encompassing four countries and a total of 5,868,660 individuals. The results show a high degree of heterogeneity among the included studies (*I*^*2*^ > 50%), which may be attributed to factors, such as the age of the study populations, types of surgeries and follow-up durations. Nevertheless, the overall summary analysis still indicates a consistent association between hysterectomy and oophorectomy and a reduced risk of breast cancer. This study conducted a comprehensive assessment of the risks associated with hysterectomy, oophorectomy, and the development of breast cancer. Our findings indicate that oophorectomy and hysterectomy have a protective effect against breast cancer, reducing the risk by 16% when compared to patients who did not undergo surgery. Hysterectomy alone was associated with a 13% reduction in breast cancer risk. Bilateral oophorectomy was found to lower the risk of breast cancer by approximately 19%. Conversely, unilateral oophorectomy had no impact on breast cancer risk. Among patients who underwent bilateral oophorectomy, postoperative hormone therapy effectively reduced the risk of breast cancer by 20%, while the absence of hormone therapy had no effect on breast cancer risk. Additionally, our investigation revealed that bilateral oophorectomy performed prior to menopause reduced the risk of breast cancer by 13%. However, the procedure had no impact on the risk of developing breast cancer when performed after menopause.

### Interpretation of findings

A meta-analysis of 38 studies was conducted to examine the long-term outcomes of hysterectomy with bilateral oophorectomy. The results revealed that women who underwent hysterectomy with bilateral oophorectomy had a reduced risk of breast cancer compared to those who did not undergo the surgery or only underwent hysterectomy [34], which is consistent with the findings of this study. Currently, there is no existing meta-analysis on the relationship between hysterectomy, oophorectomy, and breast cancer risk. Our study aimed to comprehensively evaluate this relationship by including all relevant studies. We hope that this research will provide new insights and guidance for further studies and clinical practice in the field.

The findings of this study suggest that hysterectomy and oophorectomy may be associated with a reduced risk of breast cancer, which aligns with the results reported by Robinson et al. [19] and is supported by Koch et al. [31]. This potential risk reduction could be attributed to the decrease in estrogen and progesterone levels as a result of these surgical procedures, given the crucial role these hormones play in breast cancer development [35, 36]. The removal of potentially diseased tissues during these surgeries may additionally contribute to lowering the likelihood of breast cancer occurrence [37]. However, it should be noted that contradictory findings exist in the literature [18]. A US-based study indicated a positive correlation between hysterectomy and breast cancer risk, while research conducted in Italy found no association between oophorectomy and breast cancer risk [20]. These discrepancies may arise from differences in the age ranges of the study populations, considering the significant role of estrogen in promoting proliferation of both normal and tumorous breast epithelial cells, as well as the varying estrogen levels produced at different life stages in women [38, 39]. As both hysterectomy and oophorectomy can impact estrogen levels, the risk of breast cancer development may differ accordingly. Some studies have indicated a reduced risk of breast cancer in women under the age of 45 following oophorectomy, while women over the age of 50 may experience a slight increase in risk [40]. These divergent research outcomes highlight the potential age-related disparities in the associations between hysterectomy, oophorectomy, and breast cancer risk, underscoring the need for further investigations and explorations.

This study suggests that bilateral oophorectomy is effective in reducing the risk of breast cancer, while unilateral oophorectomy does not have an impact on breast cancer risk. Oophorectomy, which involves the removal of the ovaries, significantly affects estrogen secretion in the female body [41]. Estrogen, known to stimulate the growth of breast cells and increase the risk of breast cancer [42], can be reduced to some extent by surgeries that lower estrogen levels. The majority of studies support a negative correlation between bilateral oophorectomy and breast cancer risk [19, 30, 31]. However, certain studies have shown no association between bilateral oophorectomy and the occurrence of breast cancer [11, 43]. These particular studies utilized hysterectomy as a reference, with the control group consisting of individuals who did not undergo surgery, or focused on women carrying BRCA1 and BRCA2 mutations. Additionally, some studies suggest that unilateral oophorectomy may also lower the risk of breast cancer [19, 31]. The discrepancy in findings may be attributed to the limited number of included studies and insufficient support for more recent research results. Consequently, further large-scale and long-term studies are required to validate the robustness of this conclusion.

Through a comprehensive analysis of patients who have undergone bilateral oophorectomy, we have identified hormone use after surgery as a contributing factor in the risk of breast cancer. Patients who receive hormone therapy after undergoing bilateral salpingectomy experience a decreased risk of breast cancer, while those who do not undergo hormone therapy show no discernible impact on their breast cancer risk. Hormone therapy typically encompasses the administration of estrogen and progesterone, both of which play a significant role in maintaining breast tissue health. Rational hormone therapy for patients who have undergone bilateral oophorectomy can effectively balance hormone levels in the body, thereby reducing the risk of breast cancer. Findings from a prospective cohort study suggest that refraining from combination hormone therapy can also lower the risk of breast cancer [18], potentially attributed to variances in hormone therapy dosage and types across different studies. Performing bilateral oophorectomy in premenopausal female patients can decrease the likelihood of developing breast cancer due to their typically higher hormone levels [44], which may otherwise promote breast cancer development. By undergoing bilateral oophorectomy, the presence of hormone-producing tissues can be reduced, consequently mitigating the risk of hormone-dependent breast cancer. Conversely, for postmenopausal female patients, bilateral oophorectomy may have a limited impact on the risk of breast cancer given their already decreased hormone levels. Furthermore, the age of female patients emerges as an important consideration. Premenopausal women, typically within their reproductive years, undergo greater hormonal cyclical changes, such as ovulation, menstruation, and pregnancy, thereby potentially increasing the inherent risk of breast cancer [45–47].

### Implications and limitations

This meta-analysis synthesizes evidence regarding the relationship between hysterectomy, oophorectomy, and breast cancer risk. However, it has limitations. First, due to limited data, we could not perform subgroup analyses based on factors, such as region, age, and estrogen type, which may affect the generalizability of our findings. Additionally, only cohort and case–control studies were included; future research should consider incorporating cross-sectional studies to enhance methodological diversity. The meta-analysis also lacked covariate analysis. While the included cohort studies controlled for confounding variables, caution is needed when interpreting the results. Finally, there are relatively few studies on the relationship between hysterectomy, oophorectomy, and breast cancer risk, necessitating further validation of our findings.

## Conclusions

This meta-analysis reveals a significant association between hysterectomy and oophorectomy with the risk of developing breast cancer. Nevertheless, additional research is essential to confirm and explore the underlying mechanisms of this relationship. Future studies should consider factors, such as patient age, family history, and other relevant variables, for a comprehensive understanding of the impact of these surgical interventions on breast cancer risk.

## Supplementary Information

Below is the link to the electronic supplementary material.

Supplementary file1 (DOCX 20 KB)

## Data Availability

No datasets were generated or analyzed during the current study.
