# Supplementary material for: Association between hysterectomy, oophorectomy, and risk of breast cancer: a meta-analysis
Source: Arch Gynecol Obstet. 2025 Nov 29;312(6):2031–42. doi: 10.1007/s00404-025-08179-0 (PMC12705852; doi:10.1007/s00404-025-08179-0)
Supplement: Supplementary file 1 — Supplementary file1 (DOCX 20 KB) [file 404_2025_8179_MOESM1_ESM.docx]

Table 1: PubMed

| No. | Content | Result |
| --- | --- | --- |
| #1 | Search: "Hysterectomy"[Mesh] Sort by: Most Recent | 34,589 |
| #2 | Search: ("Hysterectomy"[Mesh]) OR (Hysterectom*[Title/Abstract]) Sort by: Most Recent | 56,751 |
| #3 | Search: "Ovariectomy"[Mesh] Sort by: Most Recent | 27,827 |
| #4 | Search: ("Ovariectomy"[Mesh]) OR ((Ovariectom*[Title/Abstract]) OR (Oophorectom*[Title/Abstract])) Sort by: Most Recent | 50,473 |
| #5 | #2 OR #4 | 99,014 |
| #6 | Search: "Breast Neoplasms"[Mesh] Sort by: Most Recent | 357,415 |
| #7 | Search: ((Breast Tumor*[Title/Abstract]) OR (Breast Cancer*[Title/Abstract])) OR (Breast Carcinoma*[Title/Abstract]) Sort by: Most Recent | 390,598 |
| #8 | #6 OR #7 | 478,148 |
| #9 | #5 AND #8 | 3,875 |
| #10 | Search: "Risk"[Mesh] Sort by: Most Recent | [1,428,945](https://pubmed.ncbi.nlm.nih.gov/?sort=date&term="Risk"[Mesh]&size=200) |
| #11 | Search: ("Risk"[Mesh]) OR (risk[Title/Abstract]) Sort by: Most Recent | [3,503,517](https://pubmed.ncbi.nlm.nih.gov/?term=("Risk"[Mesh])+OR+(risk[Title/Abstract])&sort=date&size=200) |
| #12 | #9 AND #11 | 1,772 |

Table 2 Embase

| No. | Content | Result |
| --- | --- | --- |
| #1 | 'hysterectomy'/exp | 98,062 |
| #2 | hysterectom*:ab,ti | 71,602 |
| #3 | #1 OR #2 | 109,456 |
| #4 | ''oophorectomy'/exp | 64,699 |
| #5 | ovariectom*:ab,ti OR oophorectom*:ab,ti | 55,924 |
| #6 | #4 OR #5 | 81,920 |
| #7 | #3 OR #6 | 168,189 |
| #8 | 'breast tumor'/exp | 719,155 |
| #9 | 'breast cancer*':ab,ti OR 'breast carcinoma*':ab,ti OR 'breast neoplas*':ab,ti | 552,096 |
| #10 | #8 OR #9 | 777,789 |
| #11 | #7 AND #9 | 9,272 |
| #12 | 'risk'/exp | 3,282,332 |
| #13 | risk:ab,ti | 4,274,467 |
| #14 | #12 OR #13 | 5,236,469 |
| #15 | #11 AND #14 | 4,201 |

Table 3 Cochran Library

| No. | Content | Result |
| --- | --- | --- |
| #1 | MeSH descriptor: [Hysterectomy] explode all trees | 2,494 |
| #2 | (Hysterectom*):ti,ab,kw | 9,319 |
| #3 | #1OR #2 | 9,321 |
| #4 | MeSH descriptor: [Ovariectomy] explode all trees | 419 |
| #5 | (Ovariectom*):ti,ab,kw OR (Oophorectom*):ti,ab,kw | 2,391 |
| #6 | #4 OR #5 | 2,391 |
| #7 | #3 OR #6 | 10,261 |
| #8 | MeSH descriptor: [Breast Neoplasms] explode all trees | 20,356 |
| #9 | (Breast Tumor*):ti,ab,kw OR (Breast Cancer*):ti,ab,kw OR (Breast Carcinoma*):ti,ab,kw | 47,756 |
| #10 | #8 OR #9 | 48,592 |
| #11 | #7 AND #10 | 638 |
| #12 | MeSH descriptor: [Risk] explode all trees | 56,620 |
| #13 | (risk):ti,ab,kw | 320,185 |
| #14 | #12 OR #13 | 324,098 |
| #15 | #11 AND #14 | 259 |
